# Spastic Muscle Overactivity in a Patient With a Chronic Disorder of Consciousness After Severe Traumatic Brain Injury Successfully Treated with Acupuncture: A Case Report

**DOI:** 10.7759/cureus.66439

**Published:** 2024-08-08

**Authors:** Jun Matsumoto-Miyazaki, Yumiko Nishibu, Yuka Ikegame, Jun Shinoda, Hirohito Yano

**Affiliations:** 1 Chubu Medical Center for Prolonged Traumatic Brain Dysfunction, Chubu Neurorehabilitation Hospital, Minokamo, JPN; 2 Department of Cardiology, Gifu University Graduate School of Medicine, Gifu, JPN; 3 Department of Radiological Technology, Central Japan International Medical Center, Minokamo, JPN; 4 Department of Clinical Brain Sciences, Gifu University Graduate School of Medicine, Minokamo, JPN; 5 Department of Neurosurgery, Chubu Neurorehabilitation Hospital, Minokamo, JPN

**Keywords:** modified tardieu scale, ultrasound elastgraphy, disorder of consciousness, traumatic brain injury, spastic muscle overactivity, acupuncture

## Abstract

Spastic muscle overactivity (SMO) is a frequent retractable complication in patients with prolonged disorder of consciousness (DOC) after severe traumatic brain injury (sTBI). Here, we describe a patient with DOC and SMO after sTBI successfully treated with adjunctive acupuncture. A woman in her 70s with chronic DOC, corresponding to a minimally conscious state (MCS), and limb SMO after severe TBI due to a traffic accident was admitted to our hospital six months after the injury and received multiple care interventions including physiotherapy and nurse care. However, her SMO in the extremities with decorticated posture, in which bilateral elbow joints were flexed and bilateral knee joints were extended, remained. The passive range of motion (ROM) of bilateral elbow joints decreased. Her DOC also remained in MCS. Thus, acupuncture was implemented twice weekly for 20 weeks to improve SMO and promote arousal 30 months after injury. Modified Tardieu scale (MTS) during right elbow extension was measured before and after each acupuncture session. The maximum passive ROM (MTS-R2) gradually increased during the acupuncture period. The catch angle (MTS-R1), which indicates the influences of the hyper stretch reflex, also gradually reduced. The ROM limitation and the catch angle trended to decrease immediately after each acupuncture session. Ultrasound shear-wave elastography of the right brachial biceps muscle (BBM) at the 28th acupuncture session showed a reduction of shear-wave speeds immediately after the session, indicating that acupuncture reduced BBM stiffness. Her DOC state remained MCS. In the presented case, the adjunctive acupuncture therapy reduced SMO after severe TBI. Acupuncture may be beneficial for such patients. A large cohort study is warranted to confirm the effects of acupuncture on SMO in patients with DOC after sTBI.

## Introduction

Spastic muscle overactivity (SMO) is a frequent retractable complication in patients with prolonged disorder of consciousness (DOC) following severe traumatic brain injury (sTBI). Spasticity is defined as a velocity-dependent motor disorder resulting from an impaired tonic stretch reflex with exaggerated tendon reflexes and pathologic changes in the properties of rheologic muscles, including stiffness, fibrosis, and atrophy, leading to increased resistance during passive muscle movements [1,2].

Approximately 90% of patients with DOC have been reported to present with signs of spasticity, and nearly 60% demonstrated severe spasticity [3,4]. SMO has been reported to impair quality of life and increase the difficulty of rehabilitation and daily care [5-7]. SMO may sometimes interfere with the accurate assessment of DOC because of the difficulty in assessing voluntary movements that indicate consciousness [8,9]. Therefore, SMO must be treated in patients with sTBI accompanied by chronic DOC.

SMO is usually treated using oral medications including muscle relaxants, botulinum toxin injection, intrathecal baclofen therapy, and physiotherapy. In patients with stroke, acupuncture has been reported to alleviate SMO [10]. In the studies using post-stroke animal model, acupuncture has been reported to enhance the gamma amino butyric acid (GABA) pathway in the spinal cord and brain stem and alleviate spinal hyperreflexia, leading to reliving spasticity [11,12]. Previously, we reported that acupuncture reduced spinal neuron hyperexcitability in patients with DOC and SMO, suggesting that acupuncture alleviates SMO in these patients [13]. However, to the best of our knowledge, no studies have reported in detail the clinical course of SMO treated with acupuncture in patients with DOC after sTBI.

Herein, we described a patient with DOC who experienced SMO after sTBI and was successfully treated with acupuncture.

## Case presentation

**Figure 1:**
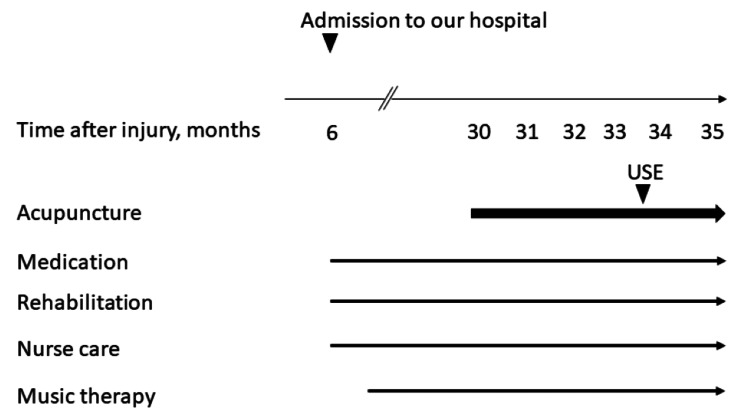
Treatment timeline Medication: levetiracetam, ursodeoxycholic acid, bisoprolol, and famotidine; Rehabilitation: rehabilitation including physiotherapy, oral care, and dysphagia rehabilitation USE: ultrasound elastography measurement

A woman patient in her 70s presented with an acute subdural hematoma and DOC after a traffic accident. She underwent craniotomy for hematoma removal since her level of consciousness was decreased (Glasgow coma scale, E2V1M2), and a ventriculoperitoneal shunt was implanted for acute hydrocephalus. Her DOC was chronic, and limb SMO developed. She was admitted to our medical center, which is a specialized hospital that treats and rehabilitates patients with DOC after sTBI due to traffic accidents, six months after the injury. Despite multiple intensive care interventions, including physiotherapy, oral care and dysphagia rehabilitation, music therapy, and nurse care, her limb SMO and DOC remained. The treatment timeline is shown in Figure 1.

**Figure 2:**
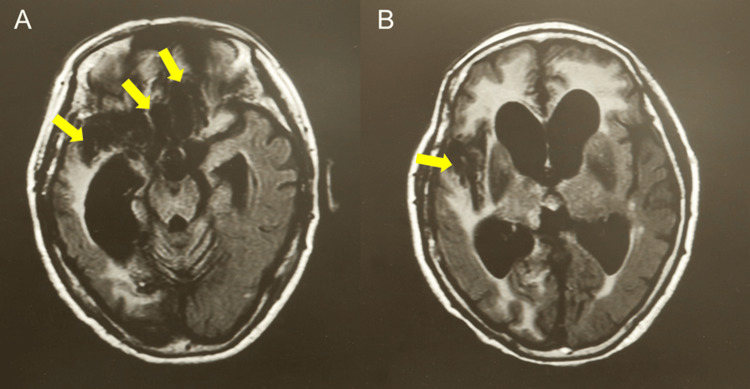
MRI FLAIR show cerebral contusions observed mainly in the bilateral frontal (A) and right temporal lobes (A and B). Yellow arrows indicate lesions of contusion. FLAIR: fluid attenuated inversion recovery

Head magnetic resonance imaging (MRI) revealed bilateral frontal and right temporal contusions (Figure 2). The patient had a minimally conscious state (MCS) minus [14,15]. At its best, spontaneous eye opening and eye pursuit were occasionally observed; however, her eyes were often closed in the daytime. Motor response to oral commands was not observed; however, voluntary finger movements of the left hand were occasionally observed. The modified Rankin scale (mRS) score was grade 5, which indicated severe disability. Glasgow outcome scale extended (GOSE) score was grade 2, which reflects a vegetative state.

She demonstrated decorticated posture due to upper and lower limb SMO, in which bilateral but predominantly right elbow joints were flexed and bilateral knee joints were extended. The passive range of motion (ROM) of elbow joint extension decreased to −85° on the right and −45° on the left.

She was prescribed levetiracetam 1000 mg/day, ursodeoxycholic acid 300 mg/day, bisoprolol 5 mg/day, and famotidine 20 mg/day. Muscle relaxants had not been prescribed because she had a past medical history of drug-induced liver dysfunction.

To improve SMO and DOC 30 months after injury, acupuncture therapy was added two times weekly for 20 weeks. Acupuncture was started using LI4, ST36, Ex-HN3, GV26, GV20, SP6, LI11, and PC6. These acupoints were selected based on previous reports, indicating the alleviation of muscle hypertonia, reduced excitability of spinal motor neuron activity [13], increased arousal and awareness [16], cerebral blood flow [17], and corticospinal tract activity [18], traditional Chinese medical theory, and our clinical experience. Acupuncture needles (0.16 or 0.20 mm in diameter and 40 mm in length) were inserted to the depth of 4-15 mm at the aforementioned acupoints according to tissue condition. A small dose of acupuncture stimulation was initially attempted and the dose was gradually increased over some weeks [19]. Acupuncture needles were retained for 10 minutes during the first session based on our previous report [13,18], and we observed no adverse events were induced by acupuncture. The time spent retaining needles was gradually increased. The needles on GV26, LI4, ST36, and PC6 were manually rotated and moved up and down every 10 minutes. The total treatment time was 50 minutes in the fourth session. Ten minutes of acupuncture in the middle of the brachial biceps muscle (BBM) as local stimulation to remarkably hypertonic muscles was added from the sixth session. ST34 and SP10 were also added for local stimulation of the femoral quadriceps (vastus lateralis and vastus medialis) from the third session. Two sessions per week of acupuncture were performed for 20 weeks, except for holidays (a total of 37 acupuncture sessions). All acupuncture sessions were performed by an acupuncturist who had a master’s degree in acupuncture and >20 years of clinical experience.

The modified Tardieu scale (MTS) score during right elbow passive extension was measured by the acupuncturist before and after each acupuncture session [20,21]. The full ROM during slow passive movement (MTS R2, max ROM), which is thought to be caused by non-stretch reflex factors, was determined using a goniometer. The angle at which resistance, a catch or clonus, was first perceived during quick passive movements (MTS R1, catch angle) was also measured [20,21]. The catch angle indicates the influence of the hyper-stretch reflex. The median values of max ROM and the catch angle before and after each acupuncture session were compared using the Wilcoxon signed-rank test.

**Figure 3:**
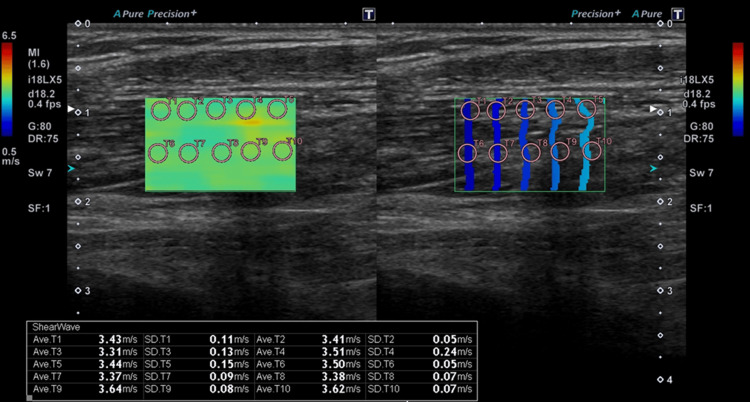
Propagation map (right) and SWS map (left) Red or yellow color on the SWS map indicates high stiffness. The pink circles show measurement ROIs of 2-mm diameter. Ten measurement ROIs on the SWS map were used for calculating SWS values after observing the quality of the contour line and color map. The mean SWS of these measurement ROIs was 3.46 m/s. SWS: shear-wave speed; ROI: region of interest

To evaluate the change in muscle stiffness, ultrasound (US) shear-wave elastography (SWE) of the right BBM was performed before and after the 28th acupuncture session by a radiological technologist with more than 15 years of clinical experience in US examination. The detailed method for US-SWE measurement was based on a previous report by the present authors [22]. Shear-wave speeds (SWS) were measured using a US system (Aplio i700; Canon Medical Systems Corporation, Otawara, Tochigi, Japan) with a linear probe (i18LX5). The patient was placed on a bed with arm relaxed. US measurements were performed twice for each elbow at 90° flexion and maximal achievable elbow extension, in which muscle contraction was not observed (−80°) [22,23]. All US images were acquired after at least five seconds of no muscle contraction. The settings of the US machine included a mechanical index of 1.3, image depth of 4 cm, scanning frequency of 18.2 MHz, spacetime (time smooth 1), dynamic range of 75 dB, single focus, tissue harmonic, and differential tissue harmonic imaging [22]. The US probe was placed parallel to the muscle fibers in the longitudinal sections of the BBM. Two-dimensional US-SWE images were acquired [22]. The shear-wave region of interest (ROI) (10 × 16 mm) for assessing BBM stiffness was set between depths of 0.5 cm and 2 cm to avoid vessels or surrounding structures (Figure 3) [22]. Ten measurement ROIs with a diameter of 2 mm were used for calculating SWS values after checking the quality of parallel wave propagation on the propagation map and color fill on the SWS map (Figure 3) [22]. Each SWS data was acquired twice and averaged [22]. Higher SWS indicated higher muscle stiffness.

The US measurements were performed before acupuncture, after 10 minutes of acupuncture needle retention on the midpoint of the BBM, and after 10 min of acupuncture needle retention on bilateral LI4, ST36, SP6, LV3, GV 26, GV20, and Ex-HN3 after the removal of the acupuncture needle on the BBM.

**Figure 4:**
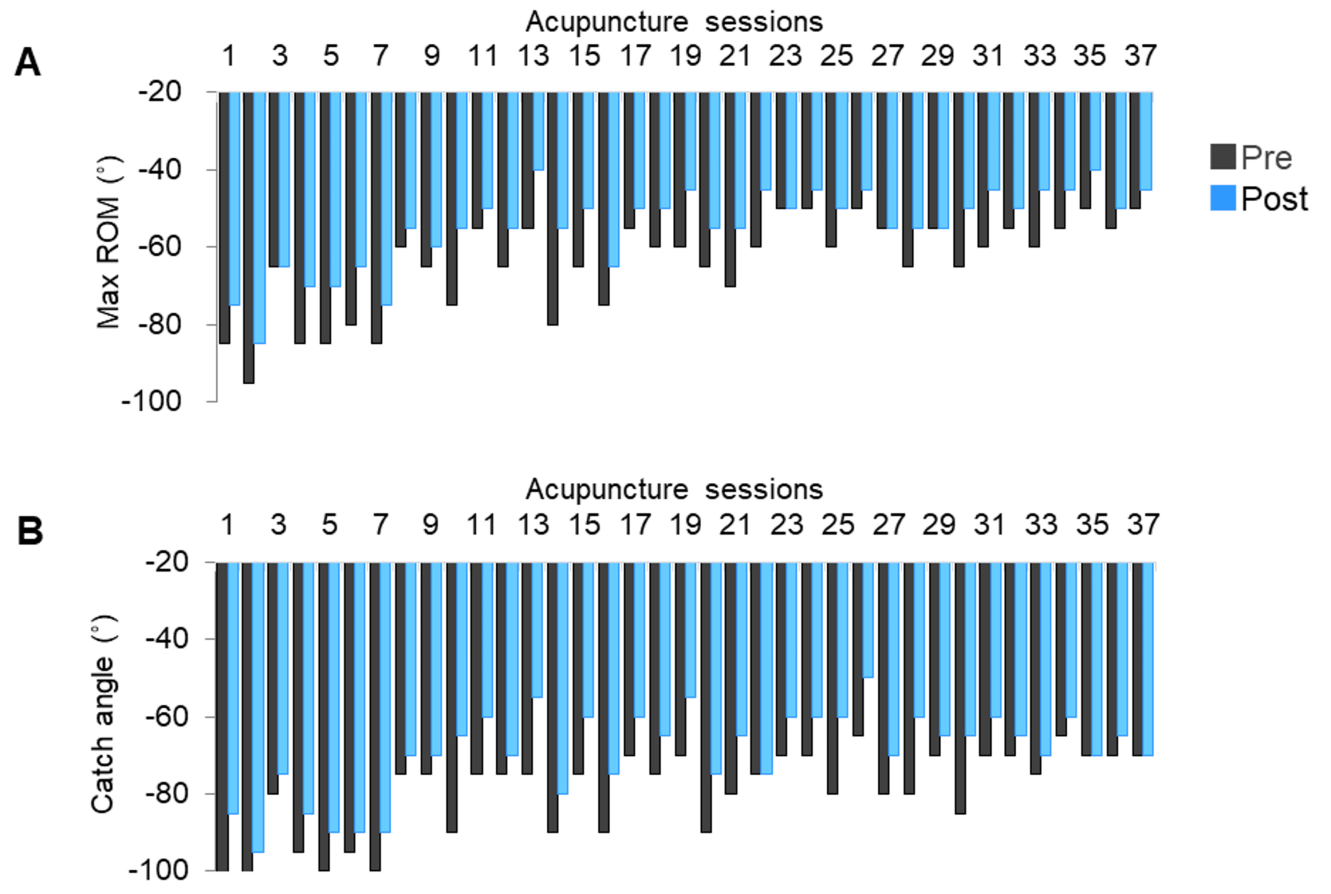
Changes in the maximum passive ROM (A) and catch angle (B) of right elbow extension before and after each acupuncture session. Black box, before acupuncture; blue box, immediately after acupuncture ROM: range of motion

The maximum passive ROM (MTS-R2) of the right elbow joint extension before each acupuncture session was gradually increased during the acupuncture period (−85° to −50°), indicating that ROM limitation improved (Figure 4A). The catch angle (MTS-R1) before each acupuncture session was also gradually reduced (Figure 4B). ROM limitation and the catch angle trended to decrease immediately after each acupuncture session (Figure 4). Significant differences were found between the median (first and third quartile) of the max ROM (−60° (−55°, −70°) vs. −50° (−45°, −55°)) and catch angle (−75° (−70°, −90°) vs. −65° (−60°, −75°)) before and immediately after acupuncture (p < 0.001, Wilcoxon signed-rank test).

**Figure 5:**
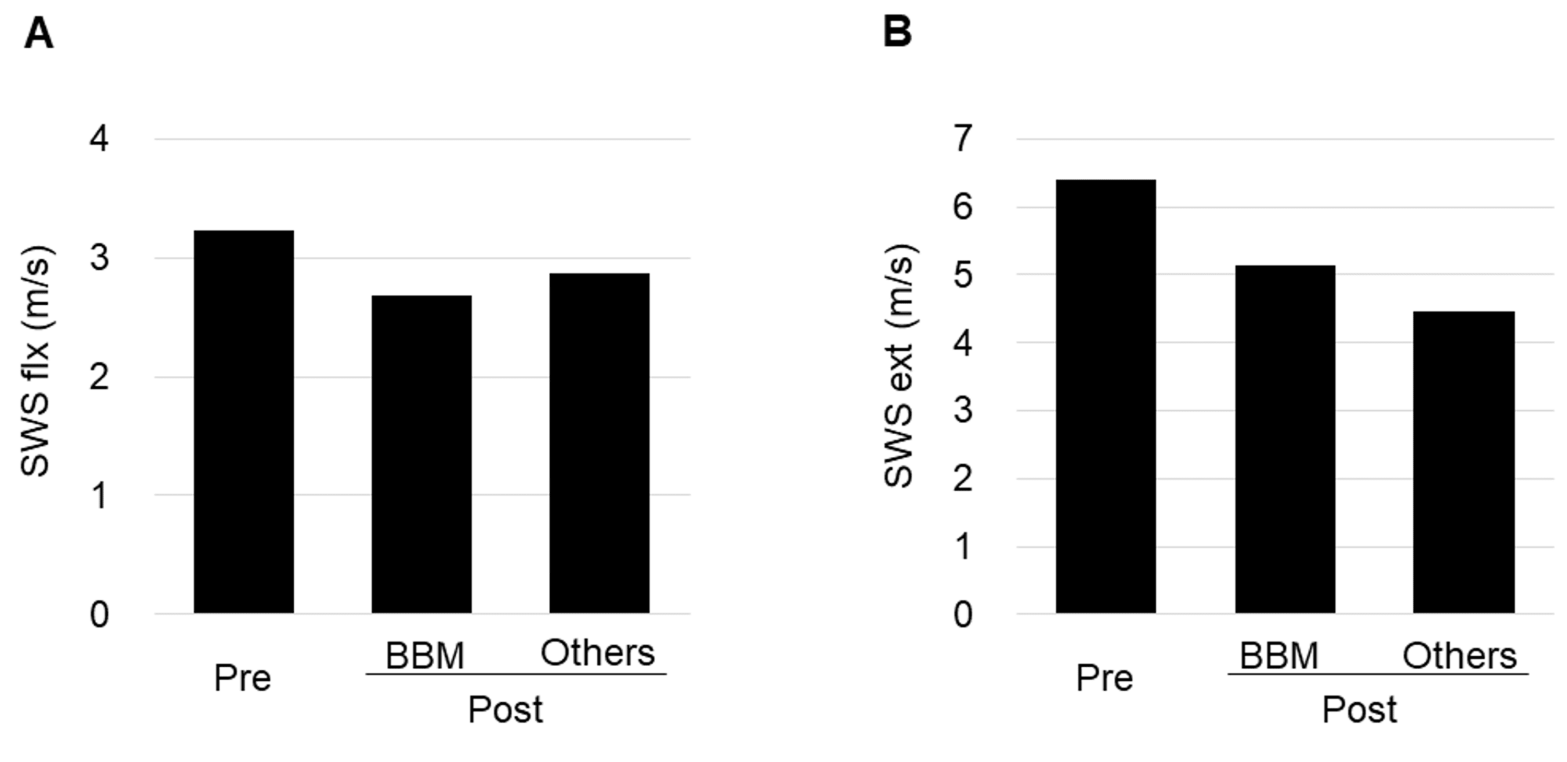
SWS in elbow flexion (A) and extension (B) before and after the 28th acupuncture session. BBM, after 10 minutes of acupuncture needle retention on the midpoint of the brachial biceps muscle; others, after 10 minutes of acupuncture needle retention on bilateral LI4, ST36, SP6, LV3, GV 20, GV26, and Ex-HN3 after the removal of the acupuncture needle on the BBM pre, before acupuncture; post, after acupuncture. The US-measured angle of elbow joint extension was −80°. BBM: brachial biceps muscle; US: ultrasound; SWS: shear-wave speeds

The SWS (elbow flexion, 3.24 m/s; maximum achievable elbow extension, 6.41 m/s) of the right BBM in the patient before acupuncture was high compared with the normal value (elbow flexion median (first, third quartile), 2.09 (1.96, 2.24) m/s; elbow extension median (first, third quartile), 3.37 (3.27, 3.94) m/s) described in the present authors' previous report [18], indicating increased muscle stiffness. The SWSs trended to decrease after acupuncture therapy compared with baseline (elbow flexion, 2.69 m/s after acupuncture on the BBM, 2.87 m/s after acupuncture on other acupoints; elbow extension, 5.15 and 4.46 m/s, respectively), suggesting that acupuncture therapy reduced BBM stiffness (Figure 5). The limited ROM of right elbow extension (before, −65°; after the BBM −55°; after other points, −55°) improved and the catch angle (−80°, −70°, −60°, respectively) reduced immediately after the same acupuncture session.

According to the impression of the medical staff including ward nurses, the duration of eye opening and time looking at the TV increased, and active movements of not only left but also right upper limbs, which were unresponsive to commands, increased after acupuncture therapy; however, these changes could not be evaluated objectively. Her DOC state was thought to remain in MCS minus. The mRS and GOSE also remained in the same grade after acupuncture started (mRS 5, GOSE 2).

No adverse events related to acupuncture occurred during the treatment period.

## Discussion

This report revealed that the limited ROM improved and the catch angle decreased during the adjunctive acupuncture period. These variables decreased immediately after each acupuncture session. BBM stiffness also decreased immediately after an acupuncture session, the max ROM improved, and the catch angle reduced. The changes over 20 weeks of adjunctive acupuncture might be induced by not only acupuncture but also other interventions, including physiotherapy and nursing care. However, given the immediate effects of acupuncture, it was suggested to reduce muscle stiffness and SMO, resulting in the improvement of ROM limitations and reduction of the catch angle. The immediate reduction in the values after acupuncture trended to increase before the next acupuncture session; however, the values before acupuncture gradually decreased throughout the acupuncture period. Therefore, repeated acupuncture in combination with other interventions such as rehabilitation might be important for long-term improvements.

The catch angle was suggested to be related to the hyperexcitability of the stretch reflex [21]. Previously, we reported that acupuncture reduced the overexcitability of the alpha motor neurons in patients with DOC and SMO after sTBI [13]. It has been reported that enhancing GABA and potassium-chloride co-transporter (KCC2) signaling pathway in the spinal cord and brain stem was related to the anti-spastic effect of acupuncture in studies using animal models of stroke [11,12]. Therefore, acupuncture might alleviate SMO in the present case by reducing the stretch reflex and alpha motor neuron overexcitability by enhancing the spinal GABA signaling pathway.

The limited ROM assessed by MTS R2 was thought to be caused by non-stretch reflex factors such as soft tissue contracture [21]. However, in the present case, alleviation of not contracture but muscle overactivity was related to the improvement of ROM limitations because immediate changes after each acupuncture session were observed.

US elastography has been used for evaluating the effects of treatment such as botulinum toxin injection on SMO in patients after a stroke [23]. Muscle stiffness assessed by SWS has been reported to correlate with the limited ROM and catch angle in patients with DOC and SMO after sTBI [22]. Recently, Yamashita et al. reported that local electric acupuncture stimulation reduced calf muscle hardness, induced by exercise, assessed using US elastography in healthy volunteers [24]. In the present case, the SWS at elbow flexion decreased immediately after local acupuncture on the BBM and did not decrease after acupuncture on other points following needle removal on the BBM. Local acupuncture stimulation on the muscle might be beneficial in reducing local muscle hardness. No study has reported changes in muscle stiffness treated with acupuncture in patients with sTBI. To our knowledge, this is the first case report using US elastography showing the immediate reduction of muscle stiffness by acupuncture in patients with DOC after sTBI.

We could not administer muscle relaxants, which are usually used for SMO, to this patient because of past history of drug-induced liver dysfunction. Acupuncture is a non-pharmacological treatment and can be used complementarily in patients even in whom pharmacological therapy is difficult.

The interpretation of the results of this report must consider some limitations. First, this is a single case report. Second, as mentioned above, the long-term alleviation of SMO during the acupuncture period was thought to be induced by not only acupuncture but also continuous rehabilitation. However, the SMO, which remained 24 months after the commencement of physiotherapy and nursing care in our hospital, had reduced after the start of acupuncture therapy. Moreover, immediate reductions in SMO were observed after each acupuncture session. Therefore, we believe that acupuncture was beneficial in alleviating SMO in the present patient. Third, MTS was measured by the acupuncturist in a clinical situation. Fourth, the US assessor was not blinded as required in a rigorous prospective study because of the nature of the clinical situation. Therefore, some biases could not be excluded. Thus, a large cohort study with blinded assessors is warranted to confirm the effects of acupuncture on the SMO in these patients.

## Conclusions

In the case of SMO after sTBI, the limited ROM improved and the catch angle was reduced during the adjunctive acupuncture period. These alleviating changes were also observed immediately after each acupuncture session. Acupuncture was seen to reduce BBM stiffness, based on US-SWE findings. Thus, acupuncture reduced SMO in the present case. Adjunctive acupuncture might be beneficial in reducing SMO after sTBI. However, a large cohort study is warranted to confirm the effects of acupuncture on SMO in patients with sTBI.
